# A Method for Reducing Training Time of ML-Based Cascade Scheme for Large-Volume Data Analysis

**DOI:** 10.3390/s24154762

**Published:** 2024-07-23

**Authors:** Ivan Izonin, Roman Muzyka, Roman Tkachenko, Ivanna Dronyuk, Kyrylo Yemets, Stergios-Aristoteles Mitoulis

**Affiliations:** 1 Department of Civil Engineering, School of Engineering, University of Birmingham, Birmingham B15 2TT, UK; s.a.mitoulis@bham.ac.uk; 2 Department of Artificial Intelligence, Lviv Polytechnic National University, 79013 Lviv, Ukraine; roman.muzyka.mknssh.2022@lpnu.ua (R.M.); kyrylo.v.yemets@lpnu.ua (K.Y.); 3 Department of Publishing Information Technologies, Lviv Polytechnic National University, 79013 Lviv, Ukraine; roman.tkachenko@gmail.com; 4 Faculty of Science & Technology, Jan Dlugosz University in Czestochowa, 42-200 Czestochowa, Poland; i.dronyuk@ujd.edu.pl

**Keywords:** machine learning, cascade scheme, training time, large data analysis, PCA, Kolmogorov–Gabor polynomial

## Abstract

We live in the era of large data analysis, where processing vast datasets has become essential for uncovering valuable insights across various domains of our lives. Machine learning (ML) algorithms offer powerful tools for processing and analyzing this abundance of information. However, the considerable time and computational resources needed for training ML models pose significant challenges, especially within cascade schemes, due to the iterative nature of training algorithms, the complexity of feature extraction and transformation processes, and the large sizes of the datasets involved. This paper proposes a modification to the existing ML-based cascade scheme for analyzing large biomedical datasets by incorporating principal component analysis (PCA) at each level of the cascade. We selected the number of principal components to replace the initial inputs so that it ensured 95% variance retention. Furthermore, we enhanced the training and application algorithms and demonstrated the effectiveness of the modified cascade scheme through comparative analysis, which showcased a significant reduction in training time while improving the generalization properties of the method and the accuracy of the large data analysis. The improved enhanced generalization properties of the scheme stemmed from the reduction in nonsignificant independent attributes in the dataset, which further enhanced its performance in intelligent large data analysis.

## 1. Introduction

In today’s digital age, data have emerged as one of the most valuable types of assets across sectors, research domains, and industries. With the exponential growth of data generation from various sources, such as social media, sensors, and transactions, the ability to extract meaningful insights from large datasets has become paramount. Large data analysis, which is processed by machine learning algorithms, has proven to be instrumental in uncovering latent patterns, predicting future trends, and driving informed decision making [1].

First, large data analysis employed by machine learning algorithms enables organizations to harness the power of big data effectively. Traditional methods of data analysis struggle to cope with the sheer volume, velocity, and variety of data being generated daily. Machine learning algorithms, however, excel in handling large datasets by automatically identifying patterns, correlations, and anomalies that may be imperceptible to human analysts [2]. Through techniques such as supervised learning, unsupervised learning, and deep learning, these algorithms can sift through vast amounts of data to extract actionable insights.

Second, the predictive capabilities of machine learning algorithms are invaluable in various domains. By analyzing historical data, these algorithms can forecast future trends, behaviors, and events with remarkable accuracy. In healthcare, for instance, predictive analytics can help to anticipate disease outbreaks, personalize treatment plans, and improve patient outcomes [3,4]. The ability to anticipate future events based on data-driven insights empowers organizations to make proactive decisions and stay ahead of the curve.

Moreover, large data analysis using machine learning algorithms facilitates personalized experiences and targeted interventions. By analyzing individual preferences, behaviors, and interactions, organizations can tailor products, services, and recommendations to specific users. This level of personalization enhances customer satisfaction, drives engagement, and fosters brand loyalty. In healthcare, personalized medicine leverages patient data to customize treatment plans and improve therapeutic outcomes.

Furthermore, large data analysis with machine learning algorithms contributes to innovation and discovery across various fields [5,6]. By uncovering hidden patterns and relationships within data, researchers can make groundbreaking discoveries, develop novel solutions, and advance scientific knowledge. In drug discovery, for example, machine learning algorithms expedite the process of screening potential drug candidates, leading to the development of new treatments for diseases [7]. The insights gleaned from large data analysis fuel innovation, drive research, and pave the way for transformative breakthroughs.

However, one of the significant challenges faced in large data analysis is the considerable time and computational resources required for training ML models, particularly in cascade schemes, where multiple models are sequentially trained [8,9,10]. In the ever-evolving landscape of machine learning (ML), the utilization of cascade schemes has emerged as a powerful strategy to tackle complex problems and extract deeper insights from data. Cascade schemes involve the sequential application of multiple ML models, with each building upon the outputs of its predecessors to address different aspects of the problem at hand [11]. In domains such as healthcare, finance, and scientific research, where the timely analysis of large datasets can lead to significant discoveries and advancements, accelerating the training of ML models holds immense potential for driving innovation and improving outcomes.

One of the most common approaches to reducing the training time of algorithms during the analysis of large datasets is employing parallel processing [12,13,14]. However, in the case of the ML-based cascade scheme, which operates in series, with each subsequent step depending on the previous one, analyzing in parallel is not feasible [15,16]. An exception could be a cascade scheme where multiple machine learning algorithms are used at each level, as in [17]. And even in this case, parallelizing the algorithm will not significantly speed up the operation of the entire method due to the sequential nature of cascade operations. Thus, deploying parallel computation faces severe limitations in addressing this challenge.

The use of hardware accelerators to further expedite the training process, which involves taking advantage of advancements in technology to push the boundaries of performance and scalability, is not always available or feasible [18]. Implementing the efficient training time of machine learning algorithms essentially boils down to using linear methods [19]. However, the latter does not always lead to a high accuracy.

In [1], the authors addressed the challenge of improving the efficiency of analyzing large datasets, particularly in their classification using PCA. They investigated how PCA, in conjunction with single machine learning algorithms, impacts the classification of large datasets. The study showcased a notable reduction in training time for individual models by reducing the input data dimensionality through PCA. Furthermore, the article explored various existing adaptations of PCA for tackling this issue. Refs. [20,21,22,23] introduce alternative methods for reducing input data dimensionality that could potentially replace PCA and offer quicker transformations. Notably, the non-iterative nature of the neural-like structure discussed in [22] contributes to this capability. However, each approach’s application should be thoroughly examined independently for its effectiveness. Specifically, it should not only aim to decrease the training time but also strive to maintain or enhance the classifier’s accuracy in analysis.

In an effort to provide solutions to the challenge, the authors of [24] developed a new ML-based cascade scheme using linear machine learning methods that demonstrated a significant increase in the accuracy of analyzing biomedical datasets. It is based on the use of linear machine learning algorithms, and the reason for the selection is their high speed. The increase in accuracy was achieved through the utilization of nonlinear input expansion. In [25], this was implemented precisely through the use of Kolmogorov–Gabor polynomials. The advantage of such a step lies in the high approximation properties of the latter. However, the main drawback is the significant increase in the duration of the training procedure. This is explained by the significant increase in the number of input attributes after applying this polynomial to the machine learning algorithm at each level of the cascade. Moreover, since cascade ensembles are hierarchical constructions [26,27] that work in series, the significant depth of the ML-based cascade scheme multiplies the duration of the training procedure. A similar approach is also applied in [25]. The authors of the paper employed one of the fastest linear machine learning methods: stochastic gradient descent. However, the issue of a significant increase in the number of attributes due to the application of the Kolmogorov–Gabor polynomial at each level of the ML-based cascade scheme remained.

In view of further pushing the boundaries of cascade modelling, this study aimed to reduce the duration of the training procedure while maintaining or improving the accuracy of the ML-based cascade scheme during the analysis of large biomedical datasets. This was achieved by reducing the dimensionality of the input data space at each level of the cascade scheme using principal component analysis (PCA). By optimizing the training process in this manner, this study aimed to enhance the efficiency and scalability of data analysis pipelines and improve the generalization properties of the method [28].

The main contributions of this paper are the results of the following actions:

- We modified the existing ML-based cascade scheme, its training, and its application algorithms to enhance the efficiency of analyzing large volumes of biomedical data by using PCA at each level of the cascade. The number of principal components that replace the original inputs of the task was chosen to ensure 95% variance coverage. This approach resulted in a significant reduction in training procedure duration of the modified scheme while preserving, and, in some cases, improved the accuracy of intelligent analysis of biomedical datasets.
- We conducted a comparison of the effectiveness of the modified ML-based cascade scheme with the existing one and found a significant reduction in its training time and a small but non-negligible improvement in the accuracy. The latter can be explained by the increase in the generalization properties of the improved scheme due to the reduction in the number of independent attributes in the large dataset processed by machine learning methods at each level of the cascade.

In the subsequent sections of this paper, we describe the methodologies employed, present experimental results and analysis, and discuss the implications of our findings.

## 2. Materials and Methods

The proposed modification discussed in this paper was based on a cascade scheme from [25]. The authors of [25] developed a new cascade approximation scheme for large tabular datasets using linear machine learning methods. Given the low accuracy of these methods, the enhancement in their performance in the proposed [25] cascade scheme was attained through the utilization of the Kolmogorov–Gabor polynomial.

The approximation of large datasets in this case is carried out implicitly using high-degree polynomials. This is achieved through the construction of a hierarchical scheme, where at each level of the cascade, a second-degree Kolmogorov–Gabor polynomial is used to model nonlinearity in the given dataset. It should be noted that at each level of the cascade, a unique subset of data is used that is randomly sampled from the overall dataset. According to the method, each subsequent level of the cascade utilizes the result of the previous level as an additional feature to expand the next input data subset. In other words, when the result of the first level is used by the ensemble (approximation by a second-degree polynomial), at the second level of the ensemble, it can implicitly obtain a fourth-order polynomial. Each subsequent level of the cascade scheme, in the case of using the quadratic Kolmogorov–Gabor polynomial, implicitly doubles the order of approximation compared with the previous one. At the same time, the number of independent attributes grows very slowly compared with the use of direct approximation by high-order Kolmogorov–Gabor polynomials [25].

Despite the significant advantages in accuracy, even the use of the quadratic Kolmogorov–Gabor polynomial substantially increases the dimensionality of the problem, especially when a large number of initial inputs of the problem [29] is analyzed. This becomes critical during the analysis of large datasets. This is because, first, the duration of the training procedure significantly increases, and second, increasing the dimensionality of the problem may deteriorate the generalization properties of the selected machine learning method, affecting the practical applicability of the method [30]. Therefore, in this paper, a modification of the cascade scheme [25] is proposed, which is intended to alleviate the aforementioned drawbacks.

The proposed novel modification of the ML-based cascade scheme for large data analysis is based on the utilization of PCA to reduce the number of nonlinearly expanded independent features at each level of the cascade scheme [25]. PCA (principal component analysis) is a dimensionality reduction method used to detect and remove correlations between variables by transforming them into a new set of variables called principal components. The detailed principles of its operation are described in [1], and expanding on these is not in the scope of this paper. The additional use of PCA at each cascade level requires additional computational resources. Moreover, PCA itself is a resource-intensive method [1]. However, in the analysis of high-dimensional datasets of large volumes, with additional application of the Kolmogorov–Gabor polynomial, which significantly increases the data dimensionality and is provided by the existing cascade structure, PCA application can greatly reduce the dimensionality of subsets at each cascade level. Practical experiments showed such a reduction by at least 10 times, even when using PCA that should ensure 95% variance. Therefore, this method provides several important advantages, especially in our case, for reducing the dimensionality of the input data space that is expanded by the aforementioned polynomial to enhance the accuracy of analyzing large datasets with linear machine learning methods. First, PCA allows for selecting principal components that best explain the variance in the data, enabling the reduction in redundancy in the data while retaining the most significant information. Second, after applying PCA, the dimensionality of the data becomes smaller, reducing the computational costs for further analysis, which is particularly relevant during the analysis of large datasets. It is these advantages that formed the basis for using PCA to modify the cascade scheme from [25].

Let us consider the training and application modes algorithms of the modified ML-based cascade scheme for solving the classification task.

### 2.1. The Training Mode Algorithm

***Step 1***. Data cleaning and preparation. Data normalization according to the chosen scaler. Split the training dataset of the large dataset into a unique number N of subsets of equal size.

***Step 2***. Formation of N levels of the cascade corresponding to the number of subsets obtained in the first step.

***Step 3***. Expansion of subset 1 using a quadratic Kolmogorov–Gabor polynomial. Apply PCA to select the number of principal components that provide 95% of the variance. Train the classifier of the first level of the cascade on the modified subset 1, as described above.

***Step 4***. Expansion of subset 2 using a quadratic Kolmogorov–Gabor polynomial. Apply PCA to select the number of principal components that provide 95% of the variance. Apply subset 2 to the trained classifiers of the first level of the cascade. Obtain the desired value and add it as a new feature to the current dataset. Expand the already enlarged subset 2 by one attribute using a quadratic Kolmogorov–Gabor polynomial. Apply PCA to select the number of principal components that provide 95% of the variance. Train the classifier of the second level of the cascade on the modified subset 2, as described above.

***Step 5***. Expansion of subset 3 using a quadratic Kolmogorov–Gabor polynomial. Apply PCA to select the number of principal components that provide 95% of the variance. Apply subset 3 to the trained classifiers of the first level of the cascade. Obtain the desired value and adding it as a new feature to the current dataset. Expand the already enlarged subset 3 by one attribute using a quadratic Kolmogorov–Gabor polynomial. Apply PCA to select the number of principal components that provide 95% of the variance. Apply subset 3 to the trained classifiers of the second level of the cascade. Obtain the desired value and adding it as another new feature to the current dataset. Expand the already enlarged subset 3 by two attributes using a quadratic Kolmogorov–Gabor polynomial. Train the classifier of the third level of the cascade on the modified subset 3 as described above.

***Step 6***. Perform steps 2–5 with all subsequent N − 1 subsets. Use the last Nth subset of data to synthesize the results of the classifier of the last level of the cascade scheme; this is the criterion for stopping the cascade operation. These values are the sought-after class markers in the method training mode.

**Figure 1:**
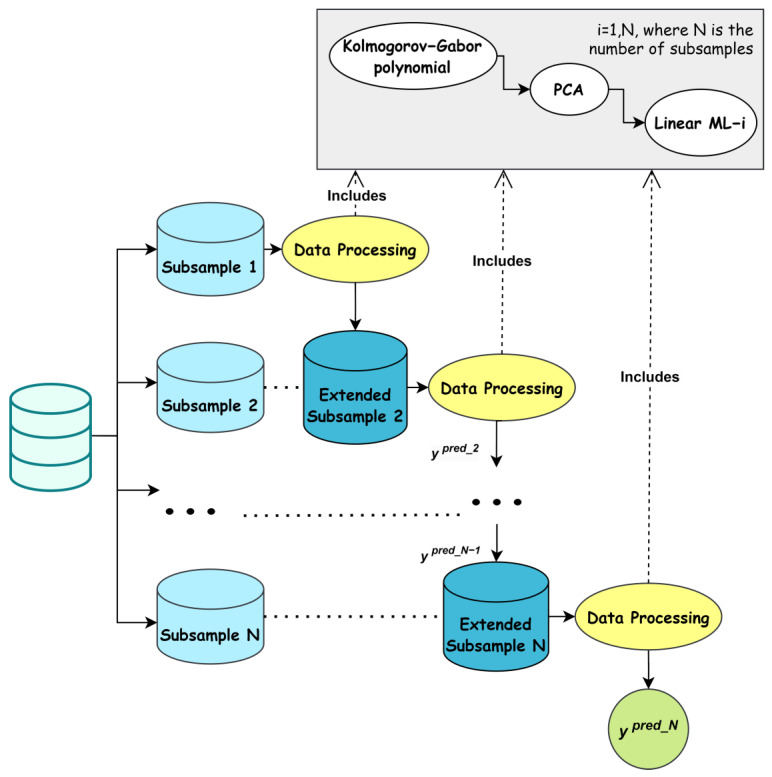
Flowchart of the modified ML-based cascade scheme for large data analysis by incorporating additional usage of PCA at each cascade level.

A flowchart of the training mode of the modified ML-based cascade scheme for large data analysis is provided in Figure 1.

### 2.2. The Application Mode Algorithm

In the application mode of the modified cascade scheme, an observation with a set of independent attributes is inputted, for which the marker of belonging to one of the defined classes must be found. This observation undergoes a series of subsequent procedures until it reaches the last level of the modified ML-based cascade scheme.

***Step 1***. Expansion of all components of the data vector with unknown output using a quadratic Kolmogorov–Gabor polynomial. Apply PCA to select the number of principal components determined in step 3 of the cascade scheme training algorithm. Apply the current vector to the trained classifier of the first level of the cascade. Obtain the desired value and add it as a new feature to the current vector.

***Step 2***. Expansion of all components of the extended data vector by one feature with an unknown output using a quadratic Kolmogorov–Gabor polynomial. Apply PCA to select the number of principal components determined in step 4 of the cascade scheme training algorithm. Apply such a vector to the trained classifier of the second level of the cascade. Obtain the desired value and add it as another new feature to the current vector.

…

***Step N***. Expansion of all components of the extended data vector, which is already expanded by N − 2 features, with unknown output using a quadratic Kolmogorov–Gabor polynomial. Apply PCA to select the number of principal components determined in step 6 of the cascade scheme training algorithm. Apply such a vector to the trained classifier of the Nth level of the cascade. Obtain the desired value, which will be the marker belonging to one of the defined classes of the task.

As a result of performing all the aforementioned steps of the application procedure, at the last level of the cascade, the desired marker belonging to one of the classes defined by the specific task can be obtained. The improvement in accuracy in this case occurs due to refining the class marker at each new level of the cascade scheme. This is achieved through the nonlinear expansion of inputs at each level of the cascade scheme by taking into account the results of the previous level. This is precisely what ensures (as demonstrated in [25]) a significant improvement in the accuracy of operation of linear, i.e., high-speed classifiers, which form the basis of the method’s operation. Additionally, the application procedure proposed in this work, which involves PCA at each level of the cascade, allows for the elimination of redundancy in the data while preserving the most significant information. This should significantly reduce the duration of the training procedure while maintaining, and in some cases even enhancing, the generalization properties and accuracy of the method overall.

## 3. Results

To model the operation of the modified ML-based cascade scheme, the authors developed custom software in Python version 1.0 [31]. The experimental analysis was conducted on a real large-scale dataset [32]. The research was conducted on the following computer: Intel^®^ Core™ i7-8750H, 2.20 GHz, and 8 GB RAM.

### 3.1. Dataset Description

Extensive datasets were obtained from the 2021 United States Disease Risk Factor Surveillance System (BRFSS) [31], which was disseminated by the Centers for Disease Control and Prevention in the United States and its surveyed regions. The parameters scrutinized by the BRFSS during the 2021 cycle encompassed health status and duration of wellness, physical exertion, hypertension screening bloc, cholesterol screening bloc, chronic illnesses, arthritic conditions, tobacco consumption, ingestion of fruits and vegetables, and access to medical assistance (principal section). Supplementary thematic modules encompassed informational domains concerning obesity and diabetes, cognitive dysfunction, patient caregiving, and post-cancer rehabilitation.

The primary dataset contained a diverse array of information regarding several aforementioned diseases. Consequently, the final dataset was refined to include only the segment of data relevant to human lifestyle factors. The primary objective was to predict occurrences of cardiovascular diseases (a binary classification task). The obtained dataset comprised 308,854 records, collectively representing 29 attributes.

### 3.2. Dataset Preprocessing

Data preprocessing consisted of two primary stages: duplicate removal and handling missing values. First, all duplicate entries were identified and eliminated to ensure the uniqueness of each record within the dataset. Subsequently, any entries with missing values within the dataset were removed. Then, the dataset was divided into training and testing subsamples, with 80% allocated to training and 20% to testing.

Following data preprocessing, the next analytical step involved addressing the class imbalance within the training part of the dataset. In the initial dataset, a class distribution ratio of 92% to 8% was observed. Balancing the classes is crucial to ensure the effective and reliable performance of the machine learning model. Class balancing within the training dataset was achieved by applying two principal algorithms in parallel: SMOTE (Synthetic Minority Oversampling Technique) to augment instances of the minority class and NearMiss to reduce instances of the majority class. This process was conducted iteratively by exploring various parameter values to adjust the number of instances [50,000 to 150,000] with a step size of Δ = 25,000 from both classes within the dataset. The accuracy and generalization were the primary criteria for selecting the appropriate balancing scheme. As demonstrated by the experiments, the optimal balancing scheme involved synthesizing 75,000 instances from each class of the original dataset. This approach precisely yielded the optimal accuracy and superior generalization properties for the classifier used.

### 3.3. Results of the Modified ML-Based Cascade Scheme Based on Its Optimal Parameters

The modified ML-based cascade scheme for large data analysis, like any other machine learning method, is characterized by a set of parameters, the optimal values of which will ensure its highest performance. Since in this study, we modified an existing cascade scheme of linear machine learning methods, one of the main parameters of its operation is the depth level of the cascade. This parameter determines how many parts the large dataset will be divided into, which influences the following:

1. The accuracy of the method’s performance.
2. The speed of the method’s operation.
3. The generalization properties of the method.

To determine the optimal value of this parameter, experimental modeling of the operation of the modified ML-based cascade scheme was conducted in this study by varying its depth from 1 to 6 levels. The accuracy of the method was evaluated using precision, recall, and F1-score performance indicators. The total accuracy was not considered, as the analysis focused on an unbalanced dataset where this metric would not provide adequate assessment of the method’s effectiveness.

It should be noted that for the modification of the method, the authors used PCA to reduce the dimensionality of the input data space at each level of the cascade, thereby reducing the duration of its training procedure. This process was automated by selecting the number of principal components at each individual level of the cascade scheme, which in sum, accounted for 95% of the variance.

**Figure 2:**
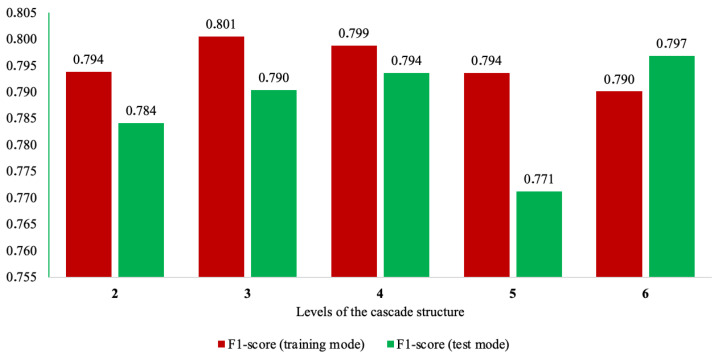
The investigation of the accuracy and generalization properties of the modified ML-based cascade scheme as its depth varied from 2 to 6 levels.

The results of this experiment based on the F1-score are presented in Figure 2.

From Figure 2, the following conclusions can be drawn:

- Increasing the number of levels of the modified ML-based cascade scheme enhanced the accuracy in both modes, but only up to a certain point. Beyond this, the method’s accuracy began to decline.
- The peak accuracy in the application mode was achieved when using a cascade scheme with four levels. However, the training mode’s accuracy for this cascade configuration was not the highest (with the difference being less than 0.02). The latter phenomenon can be explained by the fact that the four-level cascade processed data in smaller increments compared with the three-level cascade, which optimized for higher accuracy during training. While this enhances the method’s generalization properties, it may marginally reduce the training accuracy.
- The modified ML-based cascade scheme with four levels demonstrated the highest generalization properties among all other implementations. This is evident from the smallest difference in the accuracy of the method between the training and application modes.
- The use of a cascade scheme with five levels significantly deteriorated the generalization properties of the method. The application accuracy dropped by almost 3%.
- The use of a cascade scheme with six levels demonstrated overfitting. The application accuracy here surpassed the training accuracy. Therefore, further increasing the number of cascade levels did not seem appropriate.

Taking into account all the aforementioned results and considering that the four-level cascade scheme demonstrated the highest accuracy in the application mode and, most importantly, the highest generalization properties of the method, this variant of the modified ML-based cascade scheme was chosen as the final one.

In Table 1, the performance indicators of the optimized modification of the ML-based cascade scheme are summarized in both the training and application modes.

As seen from Table 1, the modified ML-based cascade scheme demonstrated high generalization across all three investigated metrics. Additionally, the duration of its training procedure, when considering the volume of input data and the four-level cascade construction, which was optimal for this task, was quite low.

The parameters from Table 1 were considered to compare the effectiveness of the modified method with a range of existing ones.

## 4. Discussion

The basic and modified versions of the ML-based cascade schemes are both based on the use of SGD (stochastic gradient descent). This choice is justified by the high speed of operation of this machine learning algorithm, which is a significant advantage when analyzing large volumes of data. Therefore, the comparison of its performance was conducted using SGD-based methods.

Thus, the effectiveness comparison of the modified ML-based cascade scheme was conducted with a range of such methods:

- Classical SGD [33] using a balanced dataset in this paper;
- Classical SGD using the Kolmogorov–Gabor polynomial [29];
- Initial ML-based cascade scheme [25].

It should be noted that the parameters of the SGD operation for all the mentioned methods above were optimized using the grid search algorithm.

**Figure 3:**
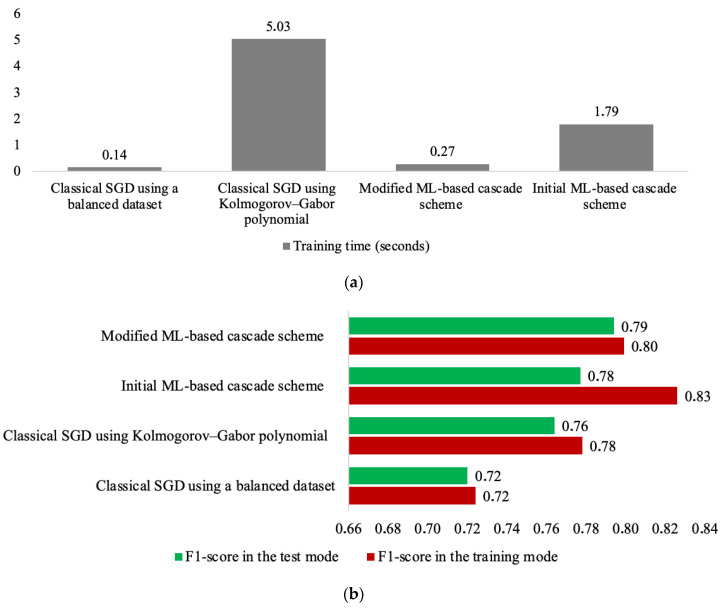
Comparison of the results for all methods investigated based on (**a**) F1-score and (**b**) training time.

Figure 3 presents the results of comparing all the methods investigated based on SGD using the F1-score and training time in seconds.

The following conclusions can be drawn based on the results of Figure 3:

- The method from [33] (classical SGD using a balanced dataset) required the shortest training time. However, this method was found to have the lowest accuracy in solving the classification task, albeit with satisfactory generalization properties.
- The method from [29] achieved higher accuracy but with poorer generalization. This was because the former employs a nonlinear input expansion based on the Kolmogorov–Gabor polynomial, which, according to Cover’s theorem, enhances the classification accuracy. However, such an algorithm with a significant expansion of inputs entails a considerable increase in the training procedure duration. Specifically, the method from [29] demonstrates an over thirty-five-fold increase in training duration compared with the method from [33].
- The initial ML-based cascade scheme from [25] demonstrated an almost threefold decrease in training duration compared with [29]. This was attributed to the significantly smaller amount of data processed by each cascade classifier. However, due to the substantial increase in the dimensionality of the task, this method showed nearly a 13-fold increase in training time compared with [33]. It is worth noting that in this case, the optimal number of cascade levels from [25] was five.
- Additionally, the method from [25] demonstrated the lowest generalization properties among all the considered methods. The difference between the training and application accuracies reached 5%. This is once again explained by the high dimensionality of the task due to the application of the Kolmogorov–Gabor polynomial at each level of the cascade scheme.
- The modified ML-based cascade scheme demonstrated a significant reduction in the training procedure duration (more than 6.6 times) compared with the base method from [25]. Thus, the goal of this article was achieved.
- Furthermore, due to the significant reduction in dimensionality of the already nonlinear input data (after applying the Kolmogorov–Gabor polynomial) through the use of PCA, it was possible to substantially improve the generalization properties of the investigated cascade scheme. Specifically, the difference between the training and application accuracy was only 1%.
- The dimensionality reduction procedure implemented in this work, which was achieved by selecting the number of principal components that accounted for 95% of the variance, enabled the automated operation of the modified ML-based cascade scheme.
- However, one of the most significant advantages of substantial dimensionality reduction in the task was that it not only preserved but also enhanced the accuracy of the modified scheme compared with the existing one. According to the F1-score, the accuracy of the modified ML-based cascade scheme was increased by 1%. This was made possible by transitioning from a large number of nonlinearly expanded inputs, as in [25], to the space of principal components and discarding more insignificant ones according to the procedure proposed in this paper. Specifically, the average number of independent attributes across all four levels of the cascade scheme after using PCA (which was intended to provide 95% variance) was 42, which were processed by the classifiers at each level. For comparison, the average number of independent variables that reached the classifiers at each level of the initial ML-based cascade scheme from [25] was 435.

Overall, the modification of the initial ML-based cascade scheme from [25] not only provided a significant reduction in training procedure duration (almost sevenfold) but also improved its generalization properties and increased the classification accuracy (by 1%). This opened up a range of advantages for the application of this method in analyzing large datasets across various domains.

In finance [34,35], where timely and accurate data analysis is crucial for decision making, the enhanced efficiency and accuracy of this modified cascade scheme could revolutionize risk assessment, portfolio management, and fraud detection processes. By reducing the training time and improving generalization, financial institutions can better handle large-scale datasets, identify market trends more swiftly, and effectively mitigate risks.

In manufacturing and engineering [36,37], the method’s ability to handle complex datasets could streamline quality control processes and predictive maintenance strategies, leading to cost savings and enhanced operational efficiency. Moreover, in fields like environmental monitoring, the method’s improved accuracy and efficiency could aid in analyzing vast amounts of data collected from sensors and satellites, thus facilitating timely interventions and policy decisions.

The versatility of this modified cascade scheme extends to the social sciences [38], where it could enhance research in areas such as sentiment analysis, demographic studies, and urban planning by providing robust tools for data-driven insights and decision making.

Among the prospects for further research, it is important to highlight efforts aimed at enhancing the accuracy of the modified ML-based cascade scheme, especially in addressing imbalanced classification tasks within the analysis of large datasets. One promising approach to achieve this could involve adopting a different data partitioning strategy, such as bootstrap sampling, for the classifiers at each cascade level. The current scheme utilizes nearly equal-sized subsets at each cascade level. Implementing bootstrap sampling, which involves generating subsets with repetitions and significantly larger volumes, would provide more diverse and informative data to each classifier in the cascade scheme, thereby potentially enhancing method accuracy.

Another research direction involves exploring alternative methods for dimensionality reduction in the input data space [20,21,22,23]. Specifically, by leveraging neural network analogs to PCA with non-iterative training [22], utilizing SGTM neural-like structures could substantially reduce the time and complexity associated with PCA at each cascade level. This enhancement would improve the efficiency of the modified cascade ensemble method in tackling diverse practical challenges.

These avenues of research hold promise for advancing the capabilities of the modified cascade ensemble approach, making it more effective in addressing various real-world applications.

## 5. Conclusions

Our study demonstrated the modification of an existing ML-based cascade scheme for analyzing large datasets through the integration of principal component analysis (PCA) at each cascade level. This highlights the efficacy of employing dimensionality reduction techniques to bolster the efficiency of cascade schemes in managing extensive data volumes.

In addition to scheme modification, we optimized the training algorithm, which ensured that the enhanced cascade operated at peak efficiency. This comprehensive approach leveraged cutting-edge techniques to derive meaningful insights from complex data.

Through rigorous comparative analysis, we assessed the effectiveness of our modified ML-based cascade scheme against the conventional approach. Our results indicate a substantial reduction in training time (over 6.6 times faster), which was accompanied by a marginal accuracy improvement (1%). These findings underscore the practical advantages of our modifications in tackling the computational complexities associated with large-scale data analysis.

The observed accuracy enhancement can be attributed to the scheme’s improved generalization properties. By reducing the number of independent attributes at each cascade level, we enhanced the scheme’s ability to generalize to unseen data, thereby boosting the overall performance in biomedical data analysis.

Looking ahead, future research will explore avenues to further optimize the cascade scheme efficiency. Specifically, we aim to investigate alternative methods for reducing the input data dimensionality, such as neural network PCA variants with non-iterative machine learning.

## Figures and Tables

**Table 1:** Results of the modified ML-based cascade scheme.

Performance Indicator	Training Mode	Test Mode
Precision	0.8	0.794
Recall	0.799	0.794
F1-score	0.799	0.794
Training time (s)	0.27	*

* means non-applicable.

## Data Availability

Publicly available datasets were used in this study. This data can be found here [32].
